# Real-world insights on nutritional awareness and behaviors among preconception and pregnant women in three Asia Pacific countries

**DOI:** 10.3389/fgwh.2024.1332555

**Published:** 2024-05-14

**Authors:** Denise Furness, Nguyen Khanh Trang Huynh, Ligaya Kaufmann, Jue Liu, Thi Bich Ngoc Nguyen, Ella Schaefer, Lucy Tan, Ching Danica Yau, Qi Yu

**Affiliations:** ^1^ Nutritionist and Molecular Geneticist, Your Genes and Nutrition, Doonan, QLD, Australia; ^2^ Department of Obstetrics and Gynaecology, Pham Ngoc Thach University of Medicine, Ho Chi Minh, Vietnam; ^3^ Department of Regulatory, Medical, Safety, Quality & Compliance (RMSQC), Bayer Consumer Care AG, Basel, Switzerland; ^4^ Department of Regulatory, Medical, Safety, Quality & Compliance (RMSQC), Bayer Healthcare Company Limited, Shanghai, China; ^5^ Department of Regulatory, Medical, Safety, Quality & Compliance (RMSQC), Bayer Vietnam Limited, Bien Hoa, Dong Nai, Vietnam; ^6^ Department of Regulatory, Medical, Safety, Quality & Compliance (RMSQC), Bayer Australia Limited, Pymble, NSW, Australia; ^7^ Department of Regulatory, Medical, Safety, Quality & Compliance (RMSQC), Bayer Healthcare Limited, Consumer Health, Hong Kong, Hong Kong SAR, China; ^8^ Gynecological Endocrinology and Reproductive Medicine Center, Department of Obstetrics and Gynecology, Peking Union Medical College Hospital, Beijing, China

**Keywords:** nutritional awareness, nutritional behaviors, preconception women, pregnant women, Asia Pacific, maternal health literacy

## Abstract

**Introduction:**

In many parts of Asia Pacific (APAC), insufficient intake of micronutrients that are important for conception and pregnancy remains a prevalent issue among women of reproductive age. It is crucial to gain insights into women's nutritional awareness and nutrition-related behaviors, as well as how these relate to their health literacy (HL). This understanding can help identify gaps and guide the development of appropriate intervention strategies. However, there appears to be limited relevant data available for the APAC region. We therefore examined nutritional awareness and behaviors among preconception and pregnant women in three APAC countries, and explored how these were related to women's HL.

**Methods:**

Cross-sectional online surveys were conducted among preconception (i.e., planning to conceive within the next 12 months or currently trying to conceive) and pregnant women in Australia (*N* = 624), China (*N* = 600), and Vietnam (*N* = 300). The survey questionnaire included a validated tool for HL (Newest Vital Sign) and questions to examine awareness and behaviors relating to healthy eating and prenatal supplementation during preconception and pregnancy.

**Results:**

Despite recommendations for a quality diet complemented by appropriate supplementation during preconception and pregnancy, many respondents in each country were not aware of the specific impact of adequate nutrition during these stages. While many respondents reported changes in their diet to eat more healthily during preconception and pregnancy, a substantial proportion were not taking prenatal supplements. Higher HL was related to greater nutritional awareness and higher use of prenatal supplements.

**Discussion:**

Our findings suggest that there are gaps in nutritional awareness and practices of many preconception or pregnant women in the three countries. Interventions to improve HL would be valuable to complement conventional knowledge-centric nutrition education, and enhance understanding and empower women to adopt appropriate nutritional practices throughout their preconception/pregnancy journey.

## 1 Introduction

Over the first 1,000 days from conception to a child's second birthday, growth and development occur more rapidly than at any other time of life (1). Throughout this early phase of life, appropriate nutrition for both mother and baby is essential to support healthy development of the fetal body and brain, and to minimize the risk of adverse outcomes and pregnancy complications (1–5). Diet quality and adequate nutritional status are also important before conception to support maternal and child health (6–8). With such evidence, it is increasingly recognized that the “window of opportunity” for successful intervention encompasses not only pregnancy but also the preconception period, and that both maternal and paternal health and nutritional status matter (6, 8–10). Over the first 1,000 days, both mother's and baby's requirements for various macronutrients and micronutrients increase and change (11–16). For parents-to-be, having a good understanding of the nutritional demands during the first 1,000 days can help them anticipate and meet these changing needs during this critical period of development, thereby establishing a solid foundation for their own health and that of their children.

**Figure 1:**
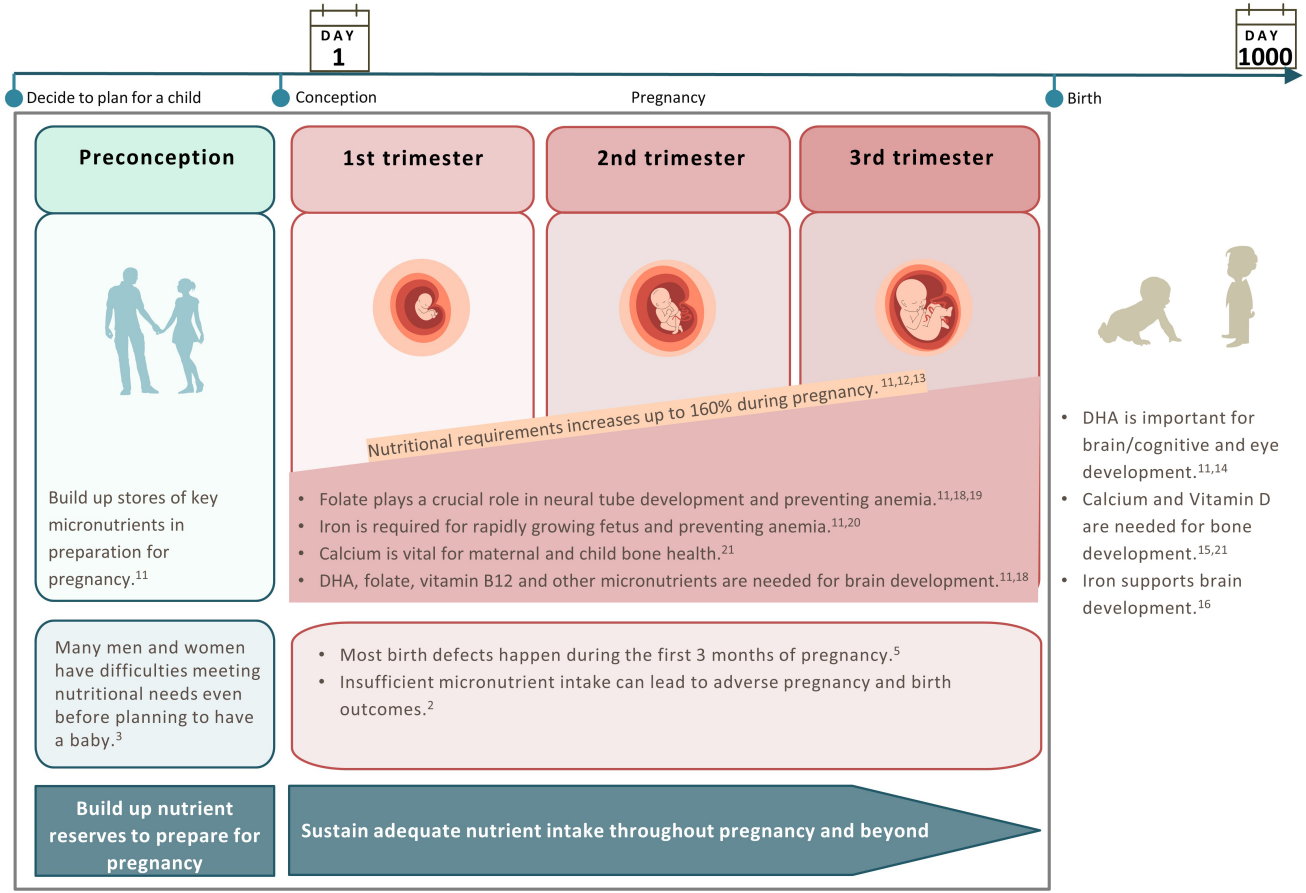
Changes in nutritional requirements to support each stage of the preconception/pregnancy journey.

Beyond meeting basic macronutrient requirements for fetal growth and development during pregnancy, it is also critical to ensure adequate intake of a range of micronutrients throughout the preconception and prenatal period as they play vital roles in fertility, fetal growth and development, and maternal and child health (Figure 1) (11). For instance, having adequate folate before pregnancy helps to support reproductive health and improve the chances of conception (17). Subsequently, in early pregnancy, folate plays a crucial role in neural tube development (11, 18). As it takes time to reach adequate folate levels during pregnancy, folate intake is considered important even before conception to minimize the risk of neural tube defects (11, 18). Throughout pregnancy, folate, iron, and vitamin B12 are all important for erythropoiesis and preventing anemia (11, 19, 20). Anemia during pregnancy adversely affects the maternal and fetal health, and is associated with increased morbidity and significant mortality risks (16). During pregnancy, calcium is vital for supporting maternal and fetal bone health, and folate, vitamin B12, and other micronutrients are needed for brain development (11, 18, 21). Suboptimal intake of key micronutrients can increase the risks of adverse maternal and fetal health outcomes, making it crucial for women to prioritize adequate nutrition before and during pregnancy (4, 8).

Increases in requirements for micronutrients during pregnancy are typically larger than the increase in energy and macronutrient requirements (11, 22). Moreover, it can take time for the body to attain the required levels of micronutrients. Therefore, having an adequate intake of critical micronutrients prior to conception helps to build up maternal nutrient stores to prepare the body for pregnancy and avoid excessive depletion and inadequacy during pregnancy (Figure 1). Besides, it is also important to sustain adequate nutrient intake throughout pregnancy to meet increased demands at this stage (Figure 1). Having a good understanding of important nutritional concepts such as building up nutrient reserves during preconception to prepare for pregnancy and sustaining adequate nutrient intake throughout pregnancy will guide parents-to-be in adopting appropriate nutritional practices. In this paper, we focus on the preconception and pregnancy stages—the crucial period where nutritional behaviors can profoundly influence child development in the first 1,000 days and maternal and child health. As there is no standard definition for the preconception stage, the working definition considered in this paper is the period from the time the decision is made to have a child up to the time of conception (Figure 1).

It is recognized that it is not always possible to obtain a sufficient amount of nutrients from the diet alone (11). Evidence suggests that use of prenatal supplements, which includes single nutrient supplements and prenatal multivitamins (PMVs), before conception and throughout pregnancy could help to address nutrient gaps from suboptimal dietary intake and positively influence pregnancy and birth outcomes (23–25). Therefore, in addition to emphasizing diet quality, appropriate supplementation is often recommended as part of preconception and prenatal care to help meet increased nutritional requirements during pregnancy (2, 11, 26).

Research on antenatal education highlights the need to go beyond simple transmission of pregnancy-related information, to address the larger issue of maternal health literacy (HL) (27). This involves equipping parents-to-be with the skills to access and analyze relevant health and nutrition information, and understand key nutritional concepts (such as building up nutrient reserves during preconception to prepare for pregnancy; sustaining adequate nutrient intake throughout pregnancy, etc.) to empower them to take actions that contribute to better health for themselves and their children. Indeed, higher maternal HL is linked to positive self-care behaviors in pregnant women and lower risks of poor health outcomes in children (28, 29).

In many parts of Asia Pacific (APAC), inadequate intake of micronutrients continues to be a prevalent issue among women in the preconception and pregnancy stages (30–33). Understanding nutritional awareness and behaviors among these women and how these relate to their HL would be helpful in identifying the gaps and informing the development of appropriate interventional strategies. However, relevant data in the APAC region appear limited (34). We therefore examined awareness of key nutritional concepts and behaviors among preconception and pregnant women who participated in a real-world research program in three APAC countries (Australia, China, and Vietnam), and explored how these were related to women's HL. Based on our observations, we highlight some key themes for maternal education in the APAC region. We also propose a concept-driven approach to nutritional education that focuses on awareness of changing nutritional needs and appropriate actions through each stage of the preconception/pregnancy journey.

## 2 Methods

### 2.1 Research design and participants

This real-world research program included cross-sectional quantitative online surveys conducted in three countries (Australia, China, and Vietnam). The data capture periods were Feb–Mar 2021 for Australia, Nov–Dec 2021 for China, and Jan–Feb 2022 for Vietnam. Data were collected by independent research organizations (Australia: fiftyfive5; China and Vietnam: IQVIA Solutions Asia) in compliance with locally applicable codes of conduct for market and social research. Ethical clearance was obtained according to local requirements. The research in Australia was approved by the Bellberry Human Research Ethics Committee (Eastwood, SA, Australia). In China and Vietnam, ethical approval was not sought since the consumer research did not involve any intervention or retrieval of data from medical records. No personal identifiable information was collected in this research.

The research participants were recruited from existing online consumer research panels selected to be representative of the included countries, and by publicizing the surveys on social media. The survey recruitment process consisted of a pre-screening step to identify potential respondents who met the eligibility criteria, and the implementation of a quota system, programmed to achieve the target sample size (number of responses) for each country. Potential respondents accessed the online survey webpage, where they provided informed consent to participate before answering the survey screening questions. Eligible respondents completed the full questionnaire. Screening questions were presented to select eligible participants, specified as follows: Women of reproductive age who had reached the legal age of marriage (18–45 years old for Australia; Vietnam or 20–45 years old for China), who were in the preconception stage (i.e., planning to conceive within the next 12 months or currently trying to conceive), or currently pregnant, and residing in specified cities in Australia, Vietnam, and China (see Supplementary Material Data S1 for a list of cities sampled in each country).

### 2.2 Questionnaire

The online survey questionnaire was provided in the main local language of each country and included custom-written questions that were created based on existing literature (35–37) to understand respondents' awareness of the importance of healthy eating and prenatal supplementation habits during preconception and pregnancy; their behavior in terms of dietary changes and supplement use during preconception and pregnancy; and sources of health and nutritional advice. The questionnaire also included a validated tool, the Newest Vital Sign (NVS), a brief measure of HL (38). The questionnaire was programmed in a way that required respondents to complete all mandatory fields.

#### 2.2.1 Newest vital sign

The NVS is a brief HL screening instrument used to assess functional HL (38). Originally developed in English and Spanish, and validated in the United States, the NVS has been adapted and validated for use in other languages and countries in general population settings (39–42), and in various groups, including caregivers of children (43) and pregnant women (44). As a brief instrument, the NVS was considered suitable for this research program due to its ease of use and acceptability to research subjects. The respondent is presented with a nutrition label from a container of ice cream, and asked six questions about the label to assess both reading and numeracy skills (38). HL levels were categorized as high likelihood of limited HL (score 0–1), possibly limited HL (score 2–3), and adequate HL (score 4–6) (38).

### 2.3 Statistical analyses

The research was designed with consideration of factors including demographics such as the size of the country's urban population, pregnancy, and birth rates for the overall population (45, 46), which informed the target sample size calculation. Based on these considerations, which determine the size of the underlying population of interest (e.g., pregnant women), the target sample sizes were derived. For Australia, *N* = 530 provided an estimated margin of error of 4% at a 95% level of confidence. Similarly, *N* = 600 provided an estimated margin of error of 4% for the survey in China, and *N* = 300 provided an estimated margin of error of 6% for the survey in Vietnam. Although the surveys were conducted by independent research organizations, each country used similar questions translated in their respective main local language. Therefore, descriptive statistics were used to summarize the responses collected in each country. Selected items were further analyzed according to stages of the preconception and pregnancy journey or HL level categories (limited HL, possibly limited HL, and adequate HL). There were no formal statistical comparisons between countries. Statistical analyses were performed using Q-research software Version 5.12.4.0 (Displayr, Chicago, IL, USA), IBM SPSS Statistics for Windows Version 25.0 (IBM Corp., Armonk, NY, USA), and WinCross Version 21.0 (The Analytical Group, Inc. Scottsdale, AZ, USA) in Australia, China, and Vietnam, respectively.

## 3 Results

### 3.1 Characteristics of respondents and health literacy levels

Respondents' characteristics are presented in Table 1. A total of 624 respondents in Australia, 600 respondents in China, and 300 respondents in Vietnam were recruited for each country. Preconception respondents made up the majority of the respondents (77%; *n* = 480) for Australia, half (50%; *n* = 300) for China and one third (33%; *n* = 100) for Vietnam. Across the three countries, most of the preconception respondents (Australia: 53%, China: 79%, Vietnam: 65%) and pregnant respondents (Australia: 61%, China: 86%, Vietnam: 85%) were between 25 and 34 years old. Most of the preconception respondents were planning to conceive within the next 12 months (Australia: 70%; China: 71%; and Vietnam: 87%). The majority of pregnant respondents reported that the current pregnancy was planned (Australia: 66%, China: 77%, Vietnam: 92%). Pregnant respondents were mostly in their first and second trimester (Australia: 58%, China: 74%, Vietnam: 83%). Around 40% of respondents in Australia and China had NVS scores 4–6 indicating adequate HL, whereas this proportion was much lower in Vietnam, with only 5% assessed to have adequate HL. More than half of the respondents (62%) in Vietnam had limited HL (NVS: 0–1).

**Table 1:** Characteristics of respondents.

Characteristics	Australia *N* = 624	China *N* = 600	Vietnam *N* = 300
Stage			
Preconception group	*n* = 480	*n* = 300	*n* = 100
Currently trying to conceive	30%	29%	13%
Planning to conceive within the next 12 months	70%	71%	87%
Age of preconception women, years			
18–24	18%	9%	10%
25–29	24%	44%	27%
30–34	29%	35%	38%
35–39	17%	10%	14%
40–45	11%	2%	11%
Pregnant group	*n* = 144	*n* = 300	*n* = 200
Trimester 1	22%	27%	36%
Trimester 2	36%	47%	47%
Trimester 3	33%	19%	18%
Not reported^a^	9%	8%	0%
Age of pregnant women, years			
18–24	18%	7%	8%
25–29	29%	55%	51%
30–34	32%	31%	34%
35–39	15%	6%	6%
40–45	6%	1%	3%
Was the pregnancy planned?			
Yes	66%	77%	92%
No	33%	22%	7%
Not reported^b^	1%	1%	1%
HL (NVS)			
Limited (0–1)	35%	22%	62%
Possibly limited (2–3)	24%	34%	33%
Adequate (4–6)	41%	44%	5%

HL, health literacy; NVS, newest vital sign.

^a^ Rather not say/Don’t know.

^b^ Rather not say.

### 3.2 Awareness of healthy eating during preconception and pregnancy

**Figure 2:**
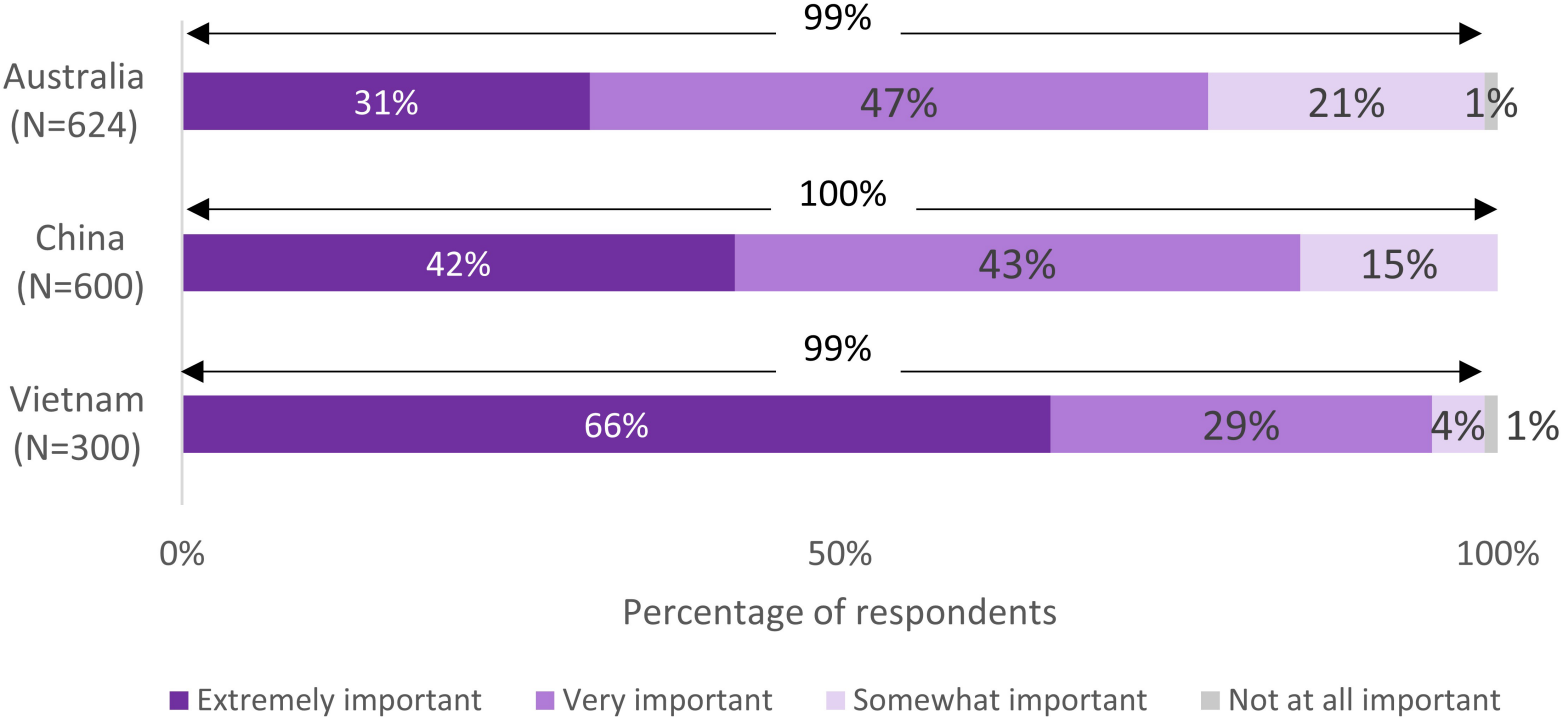
Importance of healthy eating habits during preconception and pregnancy.

**Figure 3:**
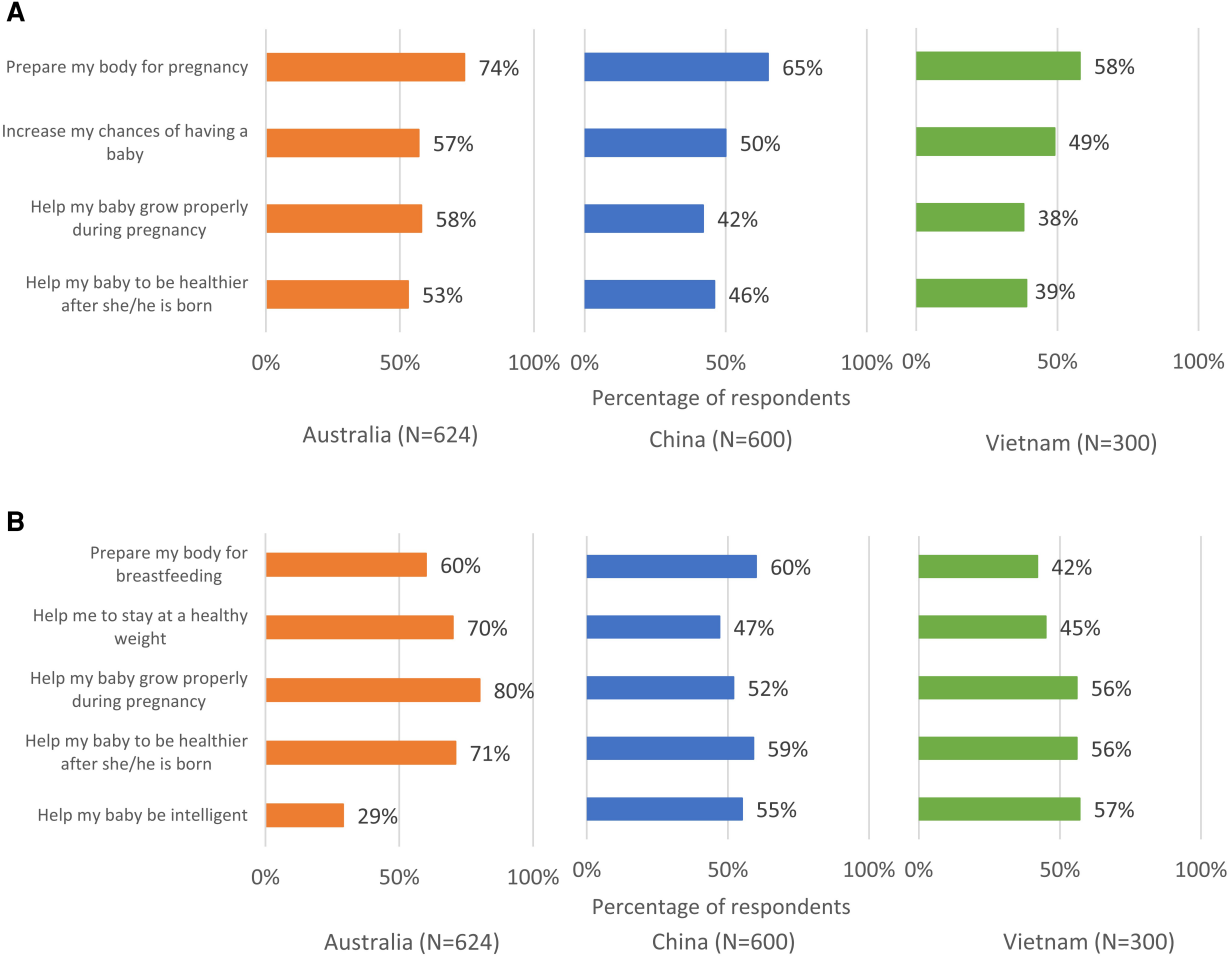
Perceived benefits of eating well when (**A**) trying to conceive and when (**B**) pregnant.

Nearly all respondents in the three countries viewed healthy eating habits during preconception and pregnancy to be somewhat, very, or extremely important (Australia: 99%, China: 100%, Vietnam: 99%) (Figure 2). However, when asked to identify specific benefits of having a healthy diet during these stages, fewer respondents in each country were aware of the key benefits at each stage (Figure 3). Up to 70% of the respondents believed that eating well before conception helps prepare the body for pregnancy (Australia: 74%, China: 65%, Vietnam: 58%). However, other benefits such as helps to increase chances of having a baby, helps baby to grow properly during pregnancy, etc., were less known (Figure 3). Most of the respondents in Australia believed that eating well during pregnancy helps the baby to grow properly during pregnancy (80%) and be healthier after birth (71%), whereas over half in China (52% and 59%, respectively) and Vietnam (56% for each benefit) were aware of these benefits. Across the countries, respondents with higher HL (including possibly limited HL or adequate HL) were more likely than those with limited HL to identify specific benefits of having a healthy diet during the stages of preconception and pregnancy (Supplementary Material Figure S1).

### 3.3 Dietary changes during preconception and pregnancy

Respondents were asked if they had made changes to the types of food and drink they consumed since becoming pregnant or planning to conceive, respectively. Many pregnant respondents in the three countries indicated they had increased their consumption of healthy foods since becoming pregnant, such as vegetables (Australia: 45%, China: 70%, Vietnam: 90%), fruits (Australia: 50%, China: 48%, Vietnam: 81%), meat/other proteins (Australia: 27%, China: 55%, Vietnam: 49%), etc., (Supplementary Material Figure S2). They reported reduced consumption of unhealthy food, such as takeaway food (Australia: 27%, China: 71%, Vietnam: 46%), sweets/desserts (Australia: 14%, China: 67%, Vietnam: 33%), alcohol (Australia: 59%, China: 70%, Vietnam: 87%), etc., (Supplementary Material Figure S2). Preconception respondents across the three countries reported a similar pattern since deciding to plan for a baby (Supplementary Material Figure S2).

### 3.4 Awareness of prenatal supplementation during preconception and pregnancy

**Figure 4:**
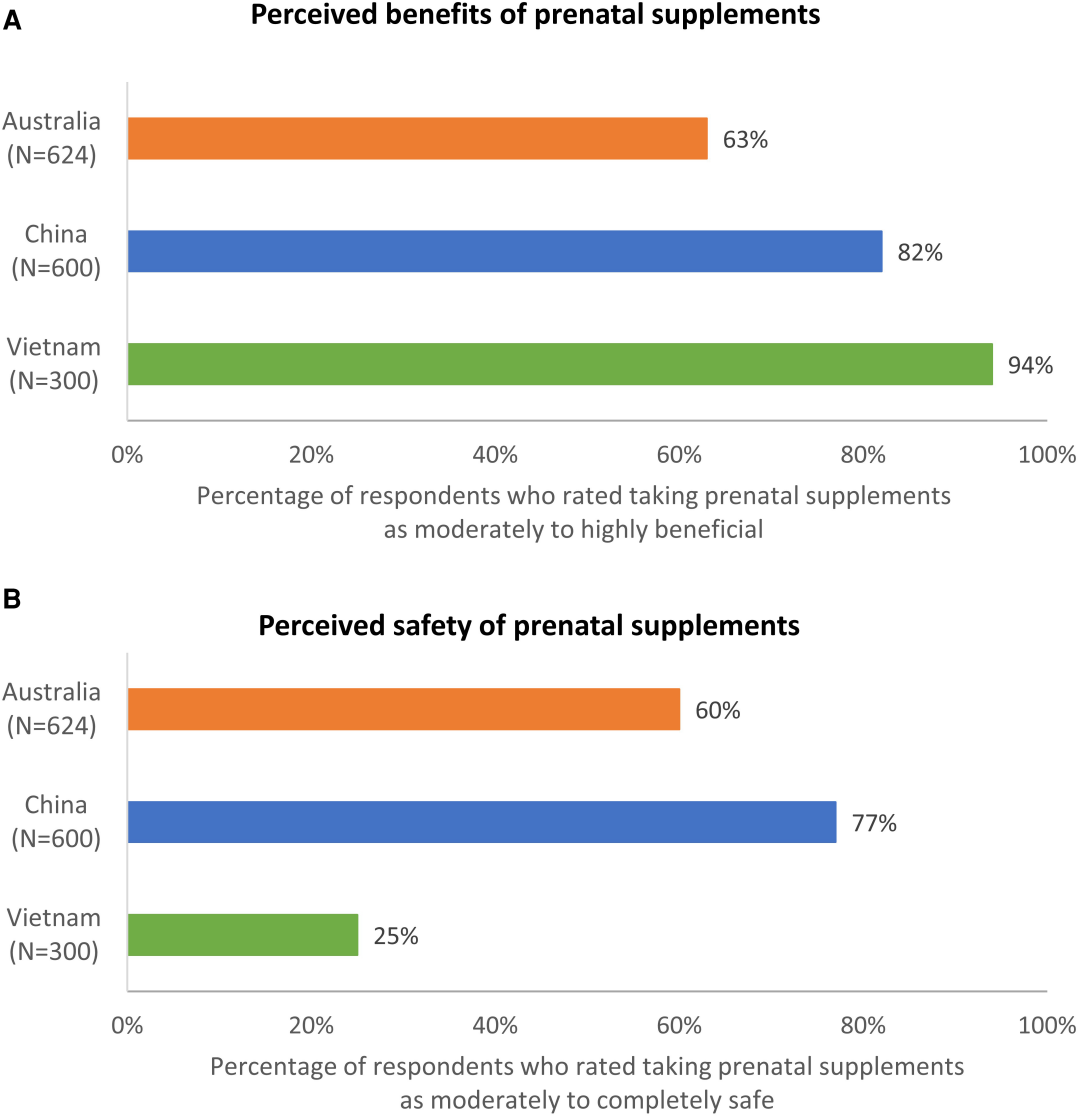
(**A**) Perceived benefit and (**B**) safety of taking prenatal supplements during preconception and pregnancy.

Respondents were asked about their perceptions of taking prenatal supplements during preconception and pregnancy in terms of both potential benefits and safety. The majority of the respondents in the three countries perceived taking supplements during these stages to be moderately to highly beneficial (Australia: 63%, China: 82%, Vietnam: 94%) (Figure 4). In Australia and China, most of the respondents regarded taking supplements to be moderately to completely safe (60% and 77%, respectively), whereas only a quarter perceived it to be so in Vietnam (25%).

**Figure 5:**
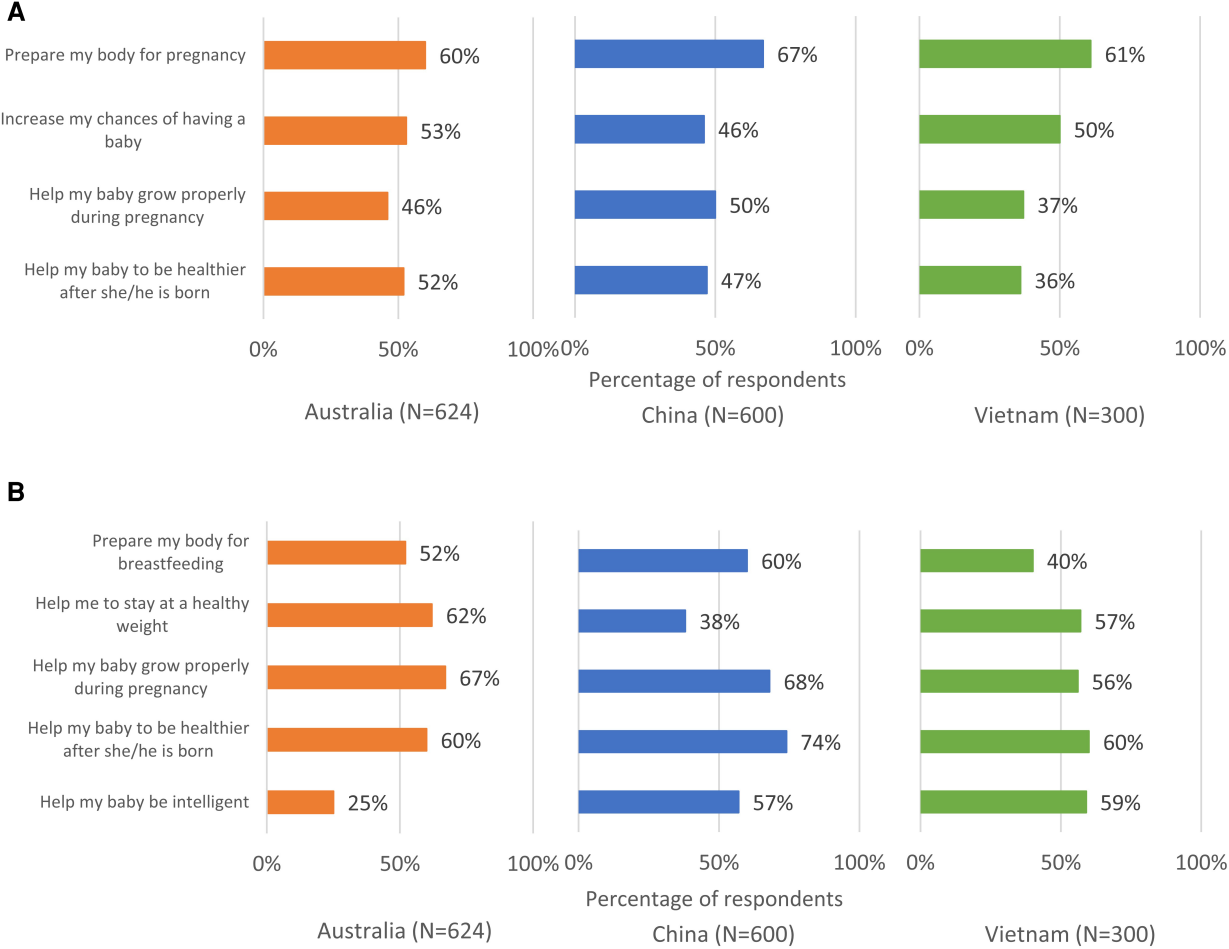
Perceived benefits of taking prenatal supplements when (**A**) trying to conceive and when (**B**) pregnant.

When asked to identify specific benefits of taking prenatal supplements during preconception and pregnancy, many respondents in each country were not aware of the key benefits during these stages (Figure 5). Only 6 in 10 were aware that taking prenatal supplements before conception could help prepare the body for pregnancy (Australia: 60%, China: 67%, Vietnam: 61%) and half believed that taking supplements before conception could increase their chances of having a baby (Australia: 53%, China: 46%, Vietnam: 50%). Across the countries, 60%–70% believed that taking supplements during pregnancy could help the baby to grow properly during pregnancy (Australia: 67%, China: 68%, Vietnam: 56%) and to be healthier after birth (Australia: 60%, China: 74%, and Vietnam: 60%). In all three countries, respondents with higher HL (including possibly limited HL or adequate HL) were more likely than those with limited HL to identify specific benefits of taking prenatal supplements during the stages of preconception and pregnancy (Supplementary Material Figure S3).

### 3.5 Supplement use during preconception and pregnancy

A substantial proportion of respondents in the three countries were not taking PMVs (Supplementary Material Table S1). Less than half of the preconception respondents who were either planning (Australia: 31%, China: 41%, Vietnam: 43%) or currently trying to conceive (Australia: 33%, China: 53%, Vietnam: 23%) were taking PMVs. Less than 65% of pregnant respondents who were in the first trimester (Australia: 52%, China: 46%, Vietnam: 30%), second trimester (Australia: 48%, China: 42%, Vietnam: 51%), or third trimester (Australia: 63%, China: 39%, Vietnam: 64%) of their pregnancy were taking PMVs. Similarly, many women were not taking other prenatal supplements such as iron and folic acid before conception and during pregnancy. Across the countries, respondents with higher HL (including possibly limited HL or adequate HL) were more likely than those with limited HL to report current use of PMVs (Table 2).

**Table 2:** Current usage of prenatal multivitamins by health literacy level.

	Australia (*N* = 624)			China (*N* = 600)			Vietnam (*N* = 300)		
	Limited HL (*n* = 220)	Possibly limited HL (*n* = 150)	Adequate HL (*n* = 254)	Limited HL (*n* = 131)	Possibly limited HL (*n* = 204)	Adequate HL (*n* = 265)	Limited HL (*n* = 186)	Possibly limited HL (*n* = 98)	Adequate HL (*n* = 16)
Percentage of respondents currently using PMVs	22%	40%	47%	30%	48%	50%	44%	43%	50%

HL, health literacy; PMVs, prenatal multivitamins.

### 3.6 Sources of information and advice on health and nutrition

In all countries, healthcare professionals (HCPs) (Australia: 61%, China: 84%, Vietnam: 77%) were regarded as the most trusted source of health-/nutrition-related information and advice among other sources, such as friend/family (Australia: 36%, China: 36%, Vietnam: 47%), websites (Australia: 32%, China: 26%, Vietnam: 36%), internet forums (Australia: 14%, China: 41%, Vietnam: 34%), etc. In Australia, both preconception respondents and pregnant respondents mostly consulted general practitioners (GPs) (55% and 54%, respectively) and midwives (51% for each) for nutritional information and advice. In China and Vietnam, obstetricians/gynecologists were most consulted by both preconception (78% and 88%, respectively) and pregnant respondents (82% and 77%, respectively). Respondents indicated that HCPs provided advice on a range of preconception/pregnancy-related issues, including dietary changes, specific nutrient requirements, and supplement use (data not shown). Respondents reported that recommendations provided by HCPs was the main reason for using prenatal supplements (data not shown).

## 4 Discussion

It is crucial for parents-to-be to understand the importance of nutrition, not just during the first 1,000 days but also prior to conception. Having a good understanding of key nutritional concepts such as building up nutrient reserves during preconception to prepare for pregnancy and sustaining adequate nutrient intake throughout pregnancy will empower parents-to-be to adopt appropriate nutritional practices to meet changing demands during this critical period of development. This real-world research program provided insights on preconception- and pregnancy-related nutritional awareness and behaviors among preconception and pregnant women in three APAC countries (Australia, China and Vietnam). It also explored how respondents' nutritional awareness and behaviors during these critical stages might be related to their levels of HL. Although many respondents in both stages reported already making positive changes to their diet, we noted gaps in awareness of specific benefits of healthy eating and prenatal supplementation, and in taking prenatal supplements in the respective stages of preconception and pregnancy. Across the three countries, respondents with higher HL level tended to be more aware of the specific benefits of eating well and prenatal supplementation in preconception and pregnancy than those with lower HL. They were also more likely than those with lower HL to use prenatal supplements. These findings highlight the need to raise women's awareness of preconception- and pregnancy-related nutritional concepts in the three countries to support them in making appropriate health-related decisions/nutritional choices during this critical period.

Respondents were aware of the general importance of eating well and the general benefits of prenatal supplementation during the preconception/pregnancy journey (Figures 2, 4A). However, they were not always able to identify specific benefits of these positive behaviors (Figures 3, 5). Approximately one quarter of the Australian respondents, a third of the Chinese respondents and close to half of the Vietnamese respondents were not aware that eating well before conception helps to prepare the body for pregnancy (Figure 3A). Other important benefits, such as eating well before conception helps to increase chances of having a baby, helps baby to grow properly during pregnancy, etc., were even less known (Figure 3A). A notable proportion of respondents across the countries were not aware that eating well during pregnancy helps the baby to grow properly during pregnancy or be healthier after birth (Figure 3B). Similar gaps were noted on the awareness of specific benefits of prenatal supplementation at each stage (Figures 5A,B). Although respondents had a general awareness of the importance of eating well and prenatal supplementation during preconception and pregnancy, they were not always able to draw connection between these behaviors to their health or that of their children. Interestingly, although the majority were aware of the general benefits of prenatal supplementation, a substantial proportion were not taking prenatal supplements during preconception/pregnancy (Figure 4A and Supplementary Material Table S1). Previous studies have also reported that although pregnant women were aware of the importance of having a healthy diet, many still did not have an optimal intake of nutrients (47–49). This is because being aware of the benefits of certain positive health behaviors (such as eating well and prenatal supplementation to support maternal and child health during preconception and pregnancy) does not necessarily translate to adopting those behaviors. This observation is highlighted in several frameworks and models of health behavior change, which recognize the influence of multiple factors on actual behavior change (50). In the present research, although a notable proportion of respondents reported that they were not supplementing during preconception/pregnancy, many reported changes in their diets to eat more healthily. There could be a misconception among the respondents that eating more healthily alone is sufficient to provide adequate nutrients to meet the demands of pregnancy; in reality, this may or may not be the case. Collectively, these observations suggest a lack of in-depth understanding of the specific benefits of adequate nutrient intake before and during pregnancy and its effects on maternal and child health, and what constitutes sufficient nutrient intake. This presents an opportunity for educating preconception and pregnant women about the key benefits of adequate nutrient intake during preconception and pregnancy.

In addition, the reported supplementation patterns suggest suboptimal awareness of the need for sustained adequate nutrient intake throughout the preconception/pregnancy journey (Supplementary Material Table S1). Rather than beginning supplementation only after pregnancy is established, starting appropriate supplementation alongside a healthy and balanced diet before conception and continue throughout pregnancy is important to derive the full benefits to meet increased nutritional demands during pregnancy (2, 11, 26). These observations suggest that respondents may not fully understand the specific impact of making positive nutritional changes early on during the preconception stage and sustaining positive behaviors throughout pregnancy.

There appeared to be greater awareness of these important preconception- and pregnancy-related nutritional concepts among respondents with higher levels of HL. This is reminiscent of observations from a recent cross-sectional survey of pregnant women in Japan (33). Healthy eating literacy scores were higher among those who had higher intake of nutrients such as iron, folic acid, calcium, DHA than those with lower intake of these nutrients (33). Our observations are also consistent with research showing that HL influences a wide range of health-related behaviors and can have a significant impact on health outcomes (51–55). Interventions to improve HL would be valuable to complement conventional knowledge-centric nutrition education and enhance women's understanding of nutritional concepts related to the respective stages of preconception and pregnancy. This can in turn empower them to adopt appropriate nutritional practices throughout their preconception/pregnancy journey.

HCPs have an important role in supporting and motivating the adoption of appropriate health-related behaviors both before conception and during pregnancy (56, 57). Our results consistently identified HCPs as the most trusted source of information for both preconception and pregnant respondents across the three countries. For Australia, midwives as well as GPs were the most important sources of nutrition advice; for China and Vietnam, this included obstetricians/gynecologists. These HCPs are well placed to help women improve their understanding of health and nutritional concepts appropriate to each stage of their parenthood journey. Our observations suggest that, despite limited consultation time, it may be valuable for HCPs to invest in making sure key nutritional concepts are well understood, with emphasis on specific benefits of doing so. These include making diet/lifestyle changes early enough in the preconception stage to build up adequate nutrient reserves for conception/pregnancy and, thereafter, the importance of consistency and sustaining positive behaviors throughout critical periods of their child's development. HCPs can tap on relevant resources and tools to support them in counselling women on preconception and maternal nutrition. For example, they could use tools such as the FIGO nutrition checklist (58) to assess women's dietary quality and identify nutritional gaps in routine visits (59, 60). Suitable training and practical guidance on preconception and pregnancy nutrition can be developed for HCPs to equip them with the relevant knowledge and skills (61). This would enable them to offer advice on the nutritional needs of both the mother and child, including the importance of balanced diets and the use of supplements (60). HCPs also need to be aware of the wide variability in HL levels among individuals, and to tailor their communications to be appropriate and accessible. HL-focused communication training may be valuable in this regard (62). Where HCPs are able to identify uncertainty or ambivalence about prenatal supplements (as evident for many respondents in Vietnam), they can proactively discuss and address these concerns. They can also provide guidance on recognizing quality supplements that have good supporting evidence on safety and effectiveness. To reinforce and complement the efforts of HCPs, it is equally critical to ensure that accurate and reliable resources and tools are widely available and accessible to women. In designing such resources, HL should also be taken into account.

A key strength of this research is that it was designed to gather data from respondents at all stages of the preconception/pregnancy journey. We aimed to extend our understanding of the current state and needs in parenthood from the preconception period through to pregnancy, which has been less studied. Another strength is that data from the three APAC countries reflect the wide range of settings across the region. This allows consideration of common themes as well as differences across the region. One of the limitations of this research was its online nature, which could have potentially introduced some bias. Individuals who choose not to participate in consumer panels or surveys, or those with limited internet use or access may be underrepresented. Due to the online nature of the data collection process, it was not possible to verify or confirm respondents' responses. Next, respondents in China and Vietnam were recruited from big cities and the results may not be generalizable to other parts of the country. As a cross-sectional research design that captured each respondent's input at a single point in time, it is not possible to use the data to understand how individuals' behavior may change over different stages of their preconception/pregnancy journey. Cohort studies specifically designed to address such questions will be needed. Nevertheless, our results are in line with the findings of earlier reports (30, 33), which suggest that many women are not supplementing before conception and during pregnancy despite recommendations for appropriate supplementation alongside a healthy and balanced diet (11, 26). NVS is a brief screening tool for assessing functional HL and does not capture all aspects of HL. For a deeper understanding of how different aspects of HL influence nutritional knowledge and behavior, studies that assess comprehensive HL may be required as a follow-up. Besides HL, other factors, such as access to maternal and child health information and services, socio-demographic factors, could also affect nutritional knowledge and behaviors. Future studies examining contributing factors of poor nutritional awareness and behaviors would be useful to improve the nutritional knowledge and practices of preconception and pregnant women. Since this research was not intended to gather data on pregnancy or birth outcomes, it is not possible to examine how HL relates to these outcomes. Longitudinal studies should be considered in future to investigate whether women with higher levels of HL are associated with better pregnancy or birth outcomes.

## 5 Conclusions

Our results indicate that many preconception or pregnant women in the three APAC countries, namely Australia, China, and Vietnam, do not completely understand key nutritional concepts of the preconception/pregnancy journey. Although there is awareness of general nutritional concepts during preconception and pregnancy, many are not aware of the specific benefits of adequate nutrition for fetal development and maternal/child health. Although most women reported making positive changes to their diet, many are not using prenatal supplements during preconception or pregnancy despite international and national recommendations for appropriate supplementation alongside a healthy and balanced diet. These observations suggest limited understanding of the concepts of making appropriate early nutritional changes during preconception and sustaining appropriate nutritional behaviors throughout pregnancy. Women with higher levels of HL show greater awareness of these important nutritional concepts for preconception/pregnancy. We propose that nutritional education efforts adopt a concept-driven approach to increase awareness of changing nutritional needs and appropriate actions through each stage of the preconception/pregnancy journey. Information delivery also needs to be tailored according to the individual's HL level. While HCPs will undoubtedly continue to play a central role in improving awareness and supporting positive behavioral change in maternity populations, it should be remembered that “it takes a village to raise a child”. Along with educational and other health-focused interventions, the support of families, communities, and society will help parents to establish adequate nutrition early on, well before conception, and continue consistently throughout the entire pregnancy to lay the foundations for their health and that of their children.

## Data Availability

The original contributions presented in the study are included in the article/**Supplementary Material**, further inquiries can be directed to the corresponding author.
