# Supplementary material for: Real-world insights on nutritional awareness and behaviors among preconception and pregnant women in three Asia Pacific countries
Source: Front Glob Womens Health. 2024 May 14;5:1332555. doi: 10.3389/fgwh.2024.1332555 (PMC11135049; doi:10.3389/fgwh.2024.1332555)
Supplement: Supplementary file 1 [file Table1.docx]

Supplementary Material

# Supplementary Data 1. List of cities sampled in each country.

Australia

Any city in Australia

China

1. Beijing
2. Guangzhou
3. Shanghai
4. Shenzhen
5. Changsha
6. Chengdu
7. Fuzhou
8. Jinan
9. Nanjing
10. Tianjin
11. Xi’an
12. Zhengzhou
13. Hefei
14. Linyi
15. Nanchang
16. Nanning
17. Shijiazhuang
18. Taiyuan
19. Weifang
20. Guiyang
21. Huaian
22. Yichang
23. Bengbu
24. Hengyang
25. Yueyang

Vietnam

1. Ho Chi Minh City
2. Hanoi
3. Da Nang
4. Can Tho

# Supplementary Figure 1. Awareness of benefits of healthy eating during preconception and pregnancy by health literacy level

#

Percentage of respondents

0

100%

0

100%

0

100%

Percentage of respondents

0

100%

0

100%

0

100%

Abbreviations: HL, health literacy

# Supplementary Figure 2 Dietary changes during preconception and pregnancy
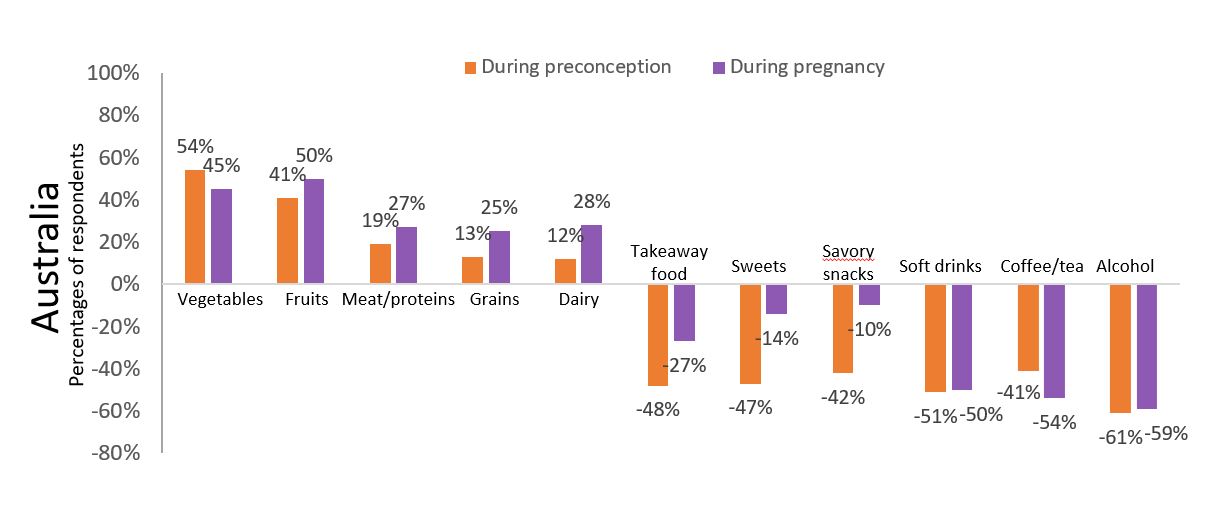

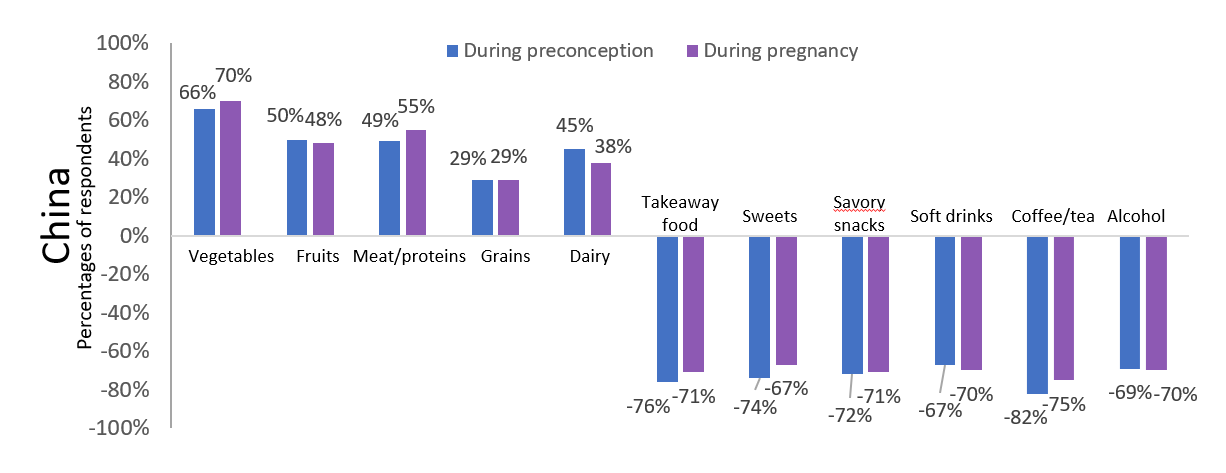

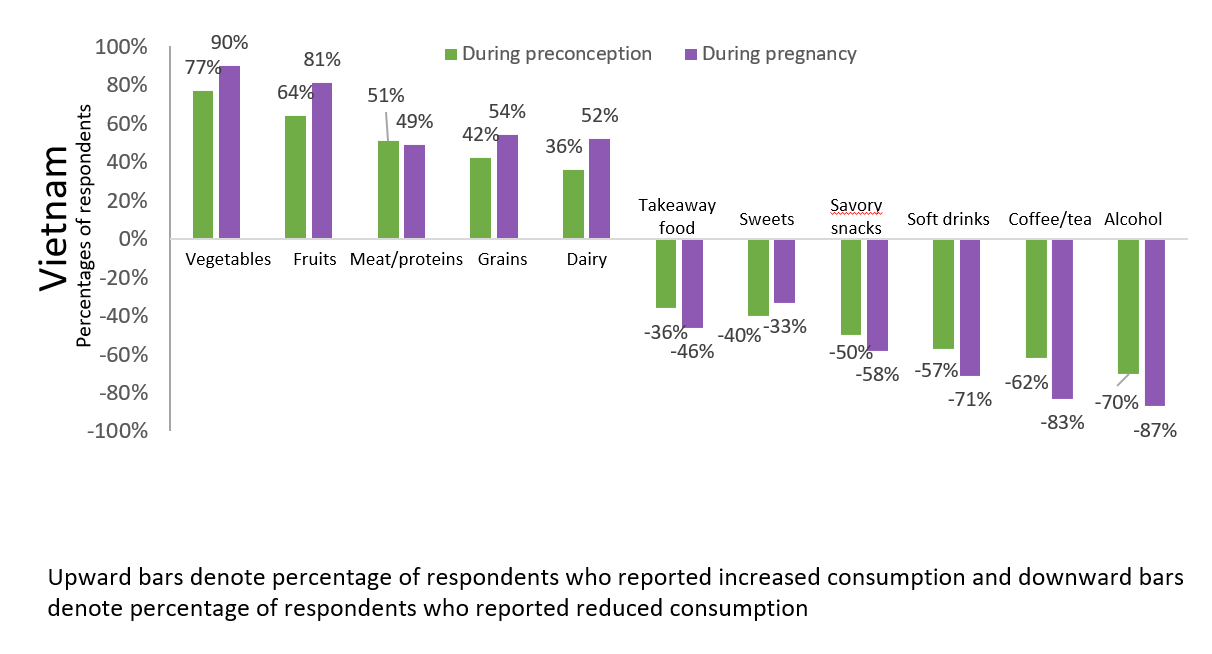


# Supplementary Figure 3 Awareness of benefits of prenatal supplementation during preconception and pregnancy by health literacy level

Percentage of respondents

0

100%

0

100%

0

100%

Percentage of respondents

0

100%

0

100%

0

100%

Abbreviations: HL, health literacy

# Supplementary Table 1 Patterns of prenatal supplement use among respondents at different stages of the preconception and pregnancy journey

| **Current use of prenatal supplements** | **Planning to conceive in next 12 months** | **Currently trying to conceive** | **Trimester 1** | **Trimester 2** | **Trimester 3** |
| --- | --- | --- | --- | --- | --- |
| n for each stage |  |  |  |  |  |
| Australia | 336 | 144 | 30 | 55 | 46 |
| China | 220 | 56 | 107 | 100 | 88 |
| Vietnam | 87 | 13 | 71 | 93 | 36 |
| PMVs |  |  |  |  |  |
| Australia | 31% | 33% | 52% | 48% | 63% |
| China | 41% | 53% | 46% | 42% | 39% |
| Vietnam | 43% | 23% | 30% | 51% | 64% |
| Iron |  |  |  |  |  |
| Australia | 25% | 23% | 34% | 33% | 64% |
| China | 13% | 20% | 19% | 22% | 30% |
| Vietnam | 53% | 77% | 54% | 66% | 72% |
| Folic acid |  |  |  |  |  |
| Australia | 28% | 34% | 36% | 27% | 26% |
| China | 40% | 52% | 42% | 38% | 34% |
| Vietnam | 33% | 31% | 24% | 18% | 47% |
| PMVs and/or folic acid |  |  |  |  |  |
| Australia | 47% | 54% | 72% | 67% | 77% |
| China | 64% | 80% | 67% | 68% | 68% |
| Vietnam | 60% | 54% | 42% | 57% | 75% |

Abbreviations: PMVs, prenatal multivitamins
